# Galactosamine and mannosamine are integral parts of bacterial and fungal extracellular polymeric substances

**DOI:** 10.1093/ismeco/ycae038

**Published:** 2024-03-22

**Authors:** Rebeca Leme Oliva, Carla Vogt, Tábata Aline Bublitz, Tessa Camenzind, Jens Dyckmans, Rainer Georg Joergensen

**Affiliations:** Soil Biology and Plant Nutrition, University of Kassel, 37213 Witzenhausen, Germany; Soil Biology and Plant Nutrition, University of Kassel, 37213 Witzenhausen, Germany; Soil Biology and Plant Nutrition, University of Kassel, 37213 Witzenhausen, Germany; Institute of Biology, Department of Plant Ecology, Freie Universität Berlin, 14195 Berlin, Germany; Centre for Stable Isotope Research Analysis, University of Göttingen, 37077 Göttingen, Germany; Soil Biology and Plant Nutrition, University of Kassel, 37213 Witzenhausen, Germany

**Keywords:** EPS, amino sugars, cell-wall components, muramic acid, glucosamine

## Abstract

Extracellular polymeric substances (EPS) are produced by microorganisms and interact to form a complex matrix called biofilm. In soils, EPS are important contributors to the microbial necromass and, thus, to soil organic carbon (SOC). Amino sugars (AS) are used as indicators for microbial necromass in soil, although the origin of galactosamine and mannosamine is largely unknown. However, indications exist that they are part of EPS. In this study, two bacteria and two fungi were grown in starch medium either with or without a quartz matrix to induce EPS production. Each culture was separated in two fractions: one that directly underwent AS extraction (containing AS from both biomass and EPS), and another that first had EPS extracted, followed then by AS determination (exclusively containing AS from EPS). We did not observe a general effect of the quartz matrix neither of microbial type on AS production. The quantified amounts of galactosamine and mannosamine in the EPS fraction represented on average 100% of the total amounts of these two AS quantified in cell cultures, revealing they are integral parts of the biofilm. In contrast, muramic acid and glucosamine were also quantified in the EPS, but with much lower contribution rates to total AS production, of 18% and 33%, respectively, indicating they are not necessarily part of EPS. Our results allow a meaningful ecological interpretation of mannosamine and galactosamine data in the future as indicators of microbial EPS, and also attract interest of future studies to investigate the role of EPS to SOC and its dynamics.

## Introduction

Extracellular polymeric substances (EPS) are produced by microorganisms, with the purpose of attaching microbial cells to each other and to surfaces [1-3]. EPS also interact to form a complex matrix often referred to as biofilm [3]. Biofilm development necessarily depends on microbial attachment, a process influenced by several factors, such as surface and cell-wall hydrophobicity as well as quantity and quality of EPS production [3]. Other than the structural function of biofilms, they also provide several strategic benefits for the microbial community, such as nutrient and water retention [2, 4, 5] and protection of extracellular enzymes [2, 6]. When biofilms are formed in soils, for example, they additionally offer a series of benefits for this ecosystem, like increasing formation and strengthening aggregate stability [7, 8], improving the soil’s drought and salinity resistance [9-11] and boosting organic matter decomposition rates [12].

Knowledge on EPS composition in soils is, however, not as advanced as in aquatic ecosystems, for instance [7]. EPS are mainly composed of exopolysaccharides and proteins, but other substances such as lipids and DNA have also been detected [2, 3, 13]. Additionally, there is growing evidence that amino sugars (AS) are also part of soil EPS [14], even though they are often not considered in the analysis of the biofilm matrix, as they are already remarked as important biomarkers for soil microbial necromass [15]. This happens mostly due to methodological constraints, involving methods used for EPS extraction [7] and lack of knowledge regarding the presence of AS in soils [14].

In soil ecosystems, AS are exclusively produced by microorganisms. Some of them form an integral part of microbial cell walls, and are used as indicators of the microbial necromass contribution to soil organic carbon (SOC) [16, 17]. Only four AS are regularly quantified in soils: muramic acid (MurN), mannosamine (ManN), galactosamine (GalN), and glucosamine (GlcN); in total contributing between 2 and 5% to SOC [18, 19]. MurN occurs exclusively in bacterial cell walls [14], especially in the murein skeleton of Gram-positive species [20, 21]. GlcN is the principal component of chitin, a polymer of N-acetyl-GlcN, in soil mainly of fungal origin [22, 23]. Furthermore, GlcN also occurs in murein, which derives from bacteria; therefore, the contribution of bacterial cell-wall murein to total soil GlcN content can be estimated, assuming that MurN and GlcN occur at a molar 1-to-2 ratio in the total amount of AS estimated in soils [20]. GlcN contributes between 47 and 68% to AS in soil, while MurN to about 3%–16% [14].

GalN is the second most common amino sugar in soil [18, 24, 25] and adds up to 42% to total AS content [14]. However, it is not a cell-wall component of bacteria, neither of actinobacteria [26] and has been rarely found in fungal [27] or archaeal cell walls [28]. Still, in bacterial and fungal laboratory cultures, 4%–15% of total AS consists of GalN [20]. Also, GalN has been previously detected as a component of teichoic acids produced by bacteria, important molecules in the mechanism of cell attachment [29, 30]. This suggests GalN occurs extracellularly, as part of the microbial residue fraction, encompassing for example EPS [14].

Lastly, ManN has been detected as a component of sialic acids in fungi, e.g. on the conidial surface of *Aspergillus fumigatus* [31] or *Gymnopilus spectabilis* [32]. ManN is additionally an unspecific marker for microbial residues (similarly to GalN), as it also has been found in bacteria, specifically in protective capsular components [33, 34] as well as in teichoic acids [35] and in EPS of Gram-positive bacteria [36]. Small amounts of ManN may also be present as linkage units between peptidoglycan and glycerol teichoic acid [37], which also indicates it could originate from microbial residues and occur in the extracellular fraction. ManN contributes approximately 4% to soil AS content [14] and due to its low concentration and specificity, ManN data have not been presented in many recent studies [16, 38-40].

It is possible that GalN and ManN are important components of EPS in soil, which has been repeatedly stated [41-44], but not yet proven. Further, the cation-exchange resin (CER) extraction method for EPS [7, 45] gives the unique possibility to directly investigate the contribution of AS, especially GalN and ManN, to freshly formed bacterial and fungal EPS under laboratory conditions. Applying this method, we tested the following hypotheses: (i) The amino sugar concentration of EPS is increased by the presence of a surface as a trigger for microbial adhesion processes. (ii) The cell-wall components MurN and GlcN remain in the biomass and contribute only small amounts to the extracted EPS. (iii) GalN and ManN are solely components of EPS and can be fully removed from the cultures by CER extraction. If successful, this would allow a meaningful ecological interpretation of ManN but especially GalN data obtained by amino sugar analysis.

## Materials and methods

### Microbial strains

Four species were chosen for this experiment for being microbes commonly found in soils and/or isolated from soils. Two were bacteria (*Escherichia coli* and *Bacillus subtilis*) and two fungi (*Fusarium acutatum* and *Arcopilus cupreus*). *B. subtilis* (DSM10) was acquired from the German Collection of Microorganisms and Cell Cultures (DSMZ) and *E. coli* (MG1655) was obtained from a culture maintained at the Freie Universität Berlin, Germany.

Both fungal strains, *F. acutatum* and *Anolis cupreus,* were isolated from an agricultural soil (unfertilized plots of the long-term field experiment in Thyrow, Humboldt-University of Berlin) and identified with the use of molecular techniques, also at the Freie Universität Berlin (details on fungal isolation can be found in Supporting information S1).

### Growth conditions for EPS production

To induce EPS production, microorganisms were individually incubated in shake flasks either with or without a matrix of SOM-free sterile quartz (SiO_2_, 0.4–0.8 mm – Carl Roth), and with a starch culture medium. This carbon source has been previously reported to yield high EPS production [46]. Starch medium contained per liter of deionized water: 45 g starch, 0.45 g yeast extract, 0.45 g peptone, 0.225 g (NH_4_)_2_SO_4_, 5.8 g KH_2_PO_4_, 2 g NaCl, 0.6 g MgSO_4_ × 7 H_2_O, and 1 ml of a micronutrient solution. The micronutrient solution used to supplement the media contained per liter of deionized water: 1.99 g FeCl_2_ × 4 H_2_O, 2.23 g MnSO_4_ × 4 H_2_O, 2.38 g CoCl_2_ × 6 H_2_O, 1.67 g CaCl_2_ × 2 H_2_O, 0.25 g CuSO_4_ × 5 H_2_O, and 0.29 g ZnSO_4_ × 7 H_2_O. Each flask was finally filled with 50 ml of culture medium, 142 g of quartz (when necessary) and the microbial species. Flasks were incubated at 30°C and shaken (100 rpm) for 4 days until EPS extraction and AS determination. After the incubation period, cultures were still in growth stage (or hyphal for the fungi) as no sporulation was detected.

### Subsampling

Following incubation, each flask was separated in two subsamples (*fraction*): (i) one that was immediately hydrolyzed for AS analysis (“cell culture”) and (ii) another that first underwent EPS extraction, only then to be hydrolyzed for AS determination (“EPS”). For the EPS extraction (resulting in the “EPS” fraction), we collected 10 ml aliquots from the cell cultures (both with and without quartz) using a standing graduated cylinder for accurate volume measurement. And for AS determination, a 2 ml aliquot was taken from either the cell cultures or the extracted EPS fractions. The purpose of this subsampling was to determine the contribution of the EPS fraction to total AS production. Assuming the “cell culture” fraction would contain AS both from the microbial cell wall (either bacterial or fungal) and from the EPS; whereas the “EPS” fraction would contain only the AS embedded in the biofilm matrix, in the extracellular environment. In this way, if the EPS contribution of an AS (MurN, ManN, GalN, or GlcN) to total AS production (quantified in the “cell culture”) was equal or higher than 90% (arbitrary threshold to avoid overestimation), for instance, that would be indication enough to assume this AS belongs to EPS (and the extracellular environment).

### EPS extraction and amino sugar analysis

The EPS extraction was carried out following the method proposed by Frølund *et al*. [45], using the recommended amount of cation exchange resin (CER) determined for *Pseudomonas putida*. The first step in this method includes a centrifugation, to ensure cells are separated from the extracellular environment [45]. Even though the CER method has been thoroughly used to extract EPS from both sludge and soil samples [7, 45, 47, 48], usually only polysaccharides and proteins are measured. Therefore, it has not been shown that this method is able to fully extract other EPS components, such as AS. One of our aims with this experiment was to test exactly that, as stated in our third hypothesis.

In order to determine AS content, the subsamples (either “cell culture” or “EPS”) were hydrolyzed in an autoclave for 10 min at 100°C (parameters determined following a hydrolysis test, described in detail in Supporting information S2). Despite the possible interference of AS from the culture medium (e.g. 0.45 g yeast extract per L, containing approximately 22.5 mg of GlcN per L, ref. 23), we chose not to use the starch medium as a blank for AS quantification. This is mainly for two reasons: first, the culture medium would not be a very accurate baseline reference as the AS (from the yeast extract) would be consumed over the course of the 4-day incubation. And secondly, because 50 ml of medium was added in each flask, meaning 1.25 mg of GlcN was initially incubated with the microbial cultures (GlcN is used as a reference because it would be the AS found in highest concentrations, Ref. 23). Assuming that part of that AS content would be degraded during incubation, we deemed this interference insignificant.

Following hydrolysis, the AS MurN, ManN, GlcN, and GalN were measured by high performance liquid chromatography (HPLC) according to Appuhn *et al*. [49] as described by Indorf *et al*. [50]. AS were automatically derivatized with ortho-phthaldialdehyde in a Dionex (Germering, Germany) HPLC Ultimate WPS-3000TSL analytical autosampler with in-line split-loop injection and thermostat, coupled to an Ultimate 3000 pump and an Ultimate 3000 fluorescence detector set at 445 nm emission and 330 nm excitation wavelengths.

### Statistical analysis

Data analysis was performed using the R Statistical Software (v4.3.1, ref. 51). Our dataset included four variables (MurN, ManN, GalN, and GlcN) and also four factors: *species* (four levels: *E. coli, B. subtilis, F. acutatum,* and *A. cupreus*), *microbial type* (two levels: bacteria and fungi), *matrix* (two levels: with and without quartz) and *fraction* (two levels: cell culture and EPS). Every quantified AS value was calculated back to the amount of cell culture it was originally extracted from (after the 4-day incubation), so results are expressed in μg of AS ml^−1^ of cell culture. We first conducted a Dixon test using the *outliers* R package (v0.15, Ref. 52) to identify potential outliers. Results with *P <* 0.05 were considered outliers and removed from the dataset. Subsequently, our data failed to pass the Shapiro–Wilk normality test (even after data transformation), carried out with the *dplyr* R package (v1.1.2, ref. 53). One reason for this was the high variation of AS quantified within different species, results partly expected. For instance, *B. subtilis* is a Gram-positive, whereas *E. coli* is a Gram-negative bacterium, which means *B. subtilis* has a much higher MurN content in its cell wall due to the thicker peptidoglycan layer [54]. Accounting for this high variation, we performed a mixed-effect linear model analysis (with the *lme4* R package - v1.1.34, Ref. 55), using *species* as a random factor to correct for the lack of normality of our dataset, whereas *microbial type*, *matrix*, and *fraction* were used as fixed factors. Results with *P* < 0.05 were considered significant effects. The data were plotted in four box plots (one per measured AS), using the SigmaPlot 13.0 software (Systat, San Jose, USA). We also calculated the contribution of EPS to total AS production (%) for each AS, using the following formula: EPS contribution (%) = AS_EPS_ / AS_CULTURE_ × 100, where AS_EPS_ represents the amount of amino sugar quantified in the EPS fraction and AS_CULTURE_ the amount quantified in the cell culture.

## Results

Matrix had no general effect alone on total amino sugar production, and the presence of quartz even led to reduced GlcN concentrations in cell cultures (Table 1, Fig. S2). Further, microbial type also did not have a significant impact on total amino sugar production as a single effect, however, when combined to other factors, bacterial and fungal species presented contrasting behaviors. For instance, regarding ManN and GalN quantification, whereas bacteria mostly had the same amounts of AS quantified both in the cell cultures and in the EPS, fungal cultures presented higher amounts of those AS quantified in the EPS when compared to cell cultures.

**Table 1:** AS quantities determined for each species and each treatment. Values are the average between replicates (*n* = 4). SD is the standard deviation between replicates and NS stands for non-significant effects (*P* > 0.05).

Species	Matrix	Suspension	MurN	ManN	GalN	GlcN
			(μg ml^−1^ of cell culture) (±SD)			
*E. coli*	Quartz	Cell culture	4 ± 0.1	8 ± 0.3	7 ± 0.01	17 ± 0.3
		EPS	0.01 ± 0.01	9 ± 0.5	7 ± 0.1	12 ± 1
	No quartz	Cell culture	9 ± 0.3	11 ± 0.1	5 ± 0.02	29 ± 1
		EPS	0.1 ± 0.06	12 ± 0.1	5 ± 0.1	15 ± 1
*B. subtillis*	Quartz	Cell culture	103 ± 5	15 ± 0.2	11 ± 0.6	115 ± 3
		EPS	42 ± 5	13 ± 0.4	8 ± 0.1	43 ± 4
	No quartz	Cell culture	111 ± 19	14 ± 1	11 ± 0.6	115 ± 19
		EPS	3 ± 1	8 ± 0.4	7 ± 0.03	14 ± 3
*F. acutatum*	Quartz	Cell culture	1 ± 0.1	3 ± 0.5	2 ± 0.2	30 ± 7
		EPS	0.1 ± 0.03	5 ± 0.01	3 ± 0.01	2 ± 0.2
	No quartz	Cell culture	2 ± 0.2	2 ± 0.3	12 ± 1	223 ± 35
		EPS	ND	4 ± 0.02	3 ± 0.02	2 ± 0.1
*A. cupreus*	Quartz	Cell culture	2 ± 0.1	9 ± 0.8	4 ± 0.6	33 ± 3
		EPS	0.6 ± 0.02	13 ± 0.4	5 ± 0.02	11 ± 1
	No quartz	Cell culture	2 ± 1	11 ± 3	3 ± 1	27 ± 17
		EPS	0.6 ± 0.1	17 ± 1	2 ± 0.05	14 ± 2
Probability values						
Matrix (quartz vs. no quartz)			NS	NS	NS	0.02
Type (bacteria vs. fungi)			NS	NS	NS	NS
Suspension (culture vs. EPS)			<0.01	NS	<0.01	<0.01
Matrix x type			NS	NS	<0.01	0.01
Matrix x suspension			NS	NS	<0.01	<0.01
Type x suspension			<0.01	<0.01	NS	NS
Matrix x type x suspension			NS	NS	<0.01	NS

ND, non-detectable value; SD, standard deviation.

Fraction, on the other hand, had significant effects in the amounts of quantified AS across all measured variables, either on its own, and/or in interaction with other factors. Moreover, even despite the fact each species produced contrasting amounts of the four AS (Table 1), the contribution of EPS to total AS production followed similar patterns across species. Regarding MurN, bacterial cell cultures presented notably higher MurN concentrations when compared to the extracted EPS. Similarly, in both matrix treatments, higher amounts of GlcN were quantified in bacterial cell cultures in comparison with the extracted EPS. The same difference was observed in the fungal cultures, however, those grown without the quartz matrix had higher GlcN production.

Bacterial cell cultures presented either the same or higher amounts of ManN when compared with the extracted EPS (Table 1, Fig. S2). Contrastingly, fungal cell cultures had less ManN quantified when compared with the extracted EPS fractions. Moreover, bacterial cell cultures also presented either equal or higher amounts of GalN compared to the extracted EPS, regardless of the presence of the matrix. Contrastingly, fungal cultures grown with quartz displayed lower GalN amounts when compared with the extracted EPS; whereas cultures grown without the matrix had higher GalN values quantified than their respective EPS fractions.

When comparing the percentage of EPS contribution to AS production (Table 2), while ManN and GalN presented an average of approximately 100% contribution of the EPS fraction, MurN and GlcN were recovered in much lower rates of 18% and 33%, respectively.

**Table 2:** Average EPS contribution to amino sugar production in cultured cells.

Species	Matrix	MurN	ManN	GalN	GlcN
		EPS contribution (%)			
*E. coli*	Quartz	0.3	113	105	69
	No quartz	1	106	102	54
*B. subtillis*	Quartz	41	85	68	38
	No quartz	2	55	65	12
*F. acutatum*	Quartz	12	194	171	8
	No quartz	ND	220	26	1
*Anolis cupreus*	Quartz	38	135	135	35
	No quartz	33	162	62	51
Average contribution (%) (±CV)		18 ± 23	134 ± 7	92 ± 6	33 ± 12

CV = mean coefficient of variation between replicates in % (*n* = 4); ND = not determined, as values were below the limit of detection.

## Discussion

The overall lack of quartz matrix effects on amino sugar production in our study refutes our first hypothesis and contrasts with what is found in the literature for other EPS components [56-58]. Biofilm formation and microbial colonization are known to be triggered by the presence of a surface, even if its effects on EPS production and composition are still not clear [59]. In this experiment, we used a matrix of sterile quartz as an inert mineral with the sole purpose to induce cell attachment. Nevertheless, it is possible that quartz’s reactivity towards microbial cells was too weak, leading to this lack of overall effect of the quartz matrix. In fact, in a study with *B. subtilis* cell cultures, when compared with other minerals such as kaolinite, montmorillonite and goethite, quartz presented significantly lower reactivity to the cells [60]. Another possibility is that the effect of a surface triggering EPS production is different for AS compared to other known EPS components such as carbohydrates and proteins, which were not measured in this study. Therefore, we encourage future experiments to test this effect in more detail with larger sample sizes and more complex experimental designs.

GalN and ManN are intrinsic parts of EPS, as expressed by the percentage of contribution of the EPS fraction to total GalN and ManN production, which was of 100% in average, supporting our second hypothesis. ManN is an amino sugar often overlooked in scientific studies. It has been rarely quantified in recent publications [16, 38-40], and when detected, values present strong variation without specificity, because ManN is produced by both fungi and bacteria [14, 26, 40]. However, ManN is a component of sialic acids, which are molecules that contribute to several cell adhesion processes [61], so it should naturally occur in EPS [14]. Our results provide some explanation to this difficulty in detecting ManN, indicating that it is an integral part of EPS. The biofilm matrix is held together by bonds with bivalent cations and hydrophobic interactions [45]. As ManN is embedded within this matrix, it is likely that the hydrolysis with 6 M HCl, (the first step to quantify AS, Ref. 50), might not be able to break all bonds within the EPS matrix on its own. In contrast, during the EPS extraction process, cell cultures are gently stirred with the CER in a step that mechanically breaks the bonds that make up the biofilm matrix, allowing for its components to be recovered later. The CER used for EPS extraction might be more efficient in breaking up the matrix and thus help to quantitatively recover ManN provided EPS extracts subsequently undergo hydrolysis with 6 M HCl [50]. This could be an explanation for the high recovery rate of ManN in the EPS, which is higher than 100%.

GalN, contrastingly, is still frequently quantified [14], and it is the second most common AS, after GlcN, found in soil and litter [18]. Our results reveal that GalN occurs exclusively in the EPS matrix, together with ManN. This observation fills a long-term knowledge gap regarding the origin of GalN [14, 44, 62]. EPS have functions similar to mucins, exuded, e.g. in the ileum of many vertebrate animals [63, 64]. These mucins are a family of proteins of high molecular weight that are heavily glycosylated and glycol-conjugated, containing high concentrations of GalN [65]. Similar mucous substances, such as EPS and capsular polysaccharides, but also lipopolysaccharides attached to the cell walls are likely the dominating source of GalN in soil [29, 66-71].

The contribution of EPS to total MurN and GlcN production was much lower (18% and 33%, respectively), indicating, that some GlcN is possibly derived from EPS. On the other hand, one could speculate that some of the MurN and GlcN extracted in the EPS fraction are due to artifacts from the extraction process itself or by the co-extraction of living and/or dead microbial cells. Microbial necromass is an important SOM pool [17] and its presence in the biofilm matrix has been previously suggested [7, 15], as dead cells could be used as nutrient source for the living biomass. We suggest that future studies investigate, for example, necromass formation within cell cultures and link it to possible indicators in the EPS matrix as a way to establish the origin of MurN and GlcN found in cultured cells EPS.

EPS as a (small) source of GlcN may be an explanation for the molar 1:2 ratio of MurN to GlcN obtained in cultured bacteria [20], which exceeds the 1:1 ratio known from textbooks. However, even though our results do not provide enough evidence to determine the origin of MurN and GlcN in the EPS fraction, our data clearly allow to dismiss them as quantitative indicators of EPS for future experiments.

As a conclusion, while muramic acid and glucosamine mostly remained in the biomass of cell cultures, galactosamine and mannosamine were fully removed by extraction with CER, indicating that the predominant (or only) source of GalN and ManN in soils are microbial EPS. Moreover, out of the total amount quantified in cell cultures, only 18% of MurN and 33% of GlcN were measured in the EPS fraction, confirming that they are cell-wall components. Further investigations are needed in order to establish why MurN and GlcN are still present in the EPS fraction and if they are indicators of microbial necromass in the biofilms. We suggest firstly the possibility that some GlcN might be part of EPS, but also that both GlcN and MurN (when found in EPS) might be indicators of microbial necromass. Our results allow for a meaningful ecological interpretation of mannosamine but especially galactosamine data in the future as indicators of EPS as part of the microbial residue fraction in soil. While there has been a strong focus on studying the contribution of necromass to SOC in recent years, this study can shift the focus to the role of EPS and other microbial residues as well. Lastly, the CER extraction method of EPS allows an improved quantitative measure of the microbial necromass contribution to SOC stocks under different environmental conditions.

## Supplementary Material

Figure_S1_ycae038

Figure_S2_ycae038

Table_S1_ISME_16_2_24_ycae038

Table_S2_ISME_16_2_24_ycae038

Table_S3_ISME_16_2_24_ycae038

## Data Availability

The datasets analyzed during the current study are available from the corresponding author upon reasonable request.
