# Supplementary figures and images for: Galactosamine and mannosamine are integral parts of bacterial and fungal extracellular polymeric substances
Source: ISME Commun. 2024 Mar 22;4(1):ycae038. doi: 10.1093/ismeco/ycae038 (PMC11014887; doi:10.1093/ismeco/ycae038)

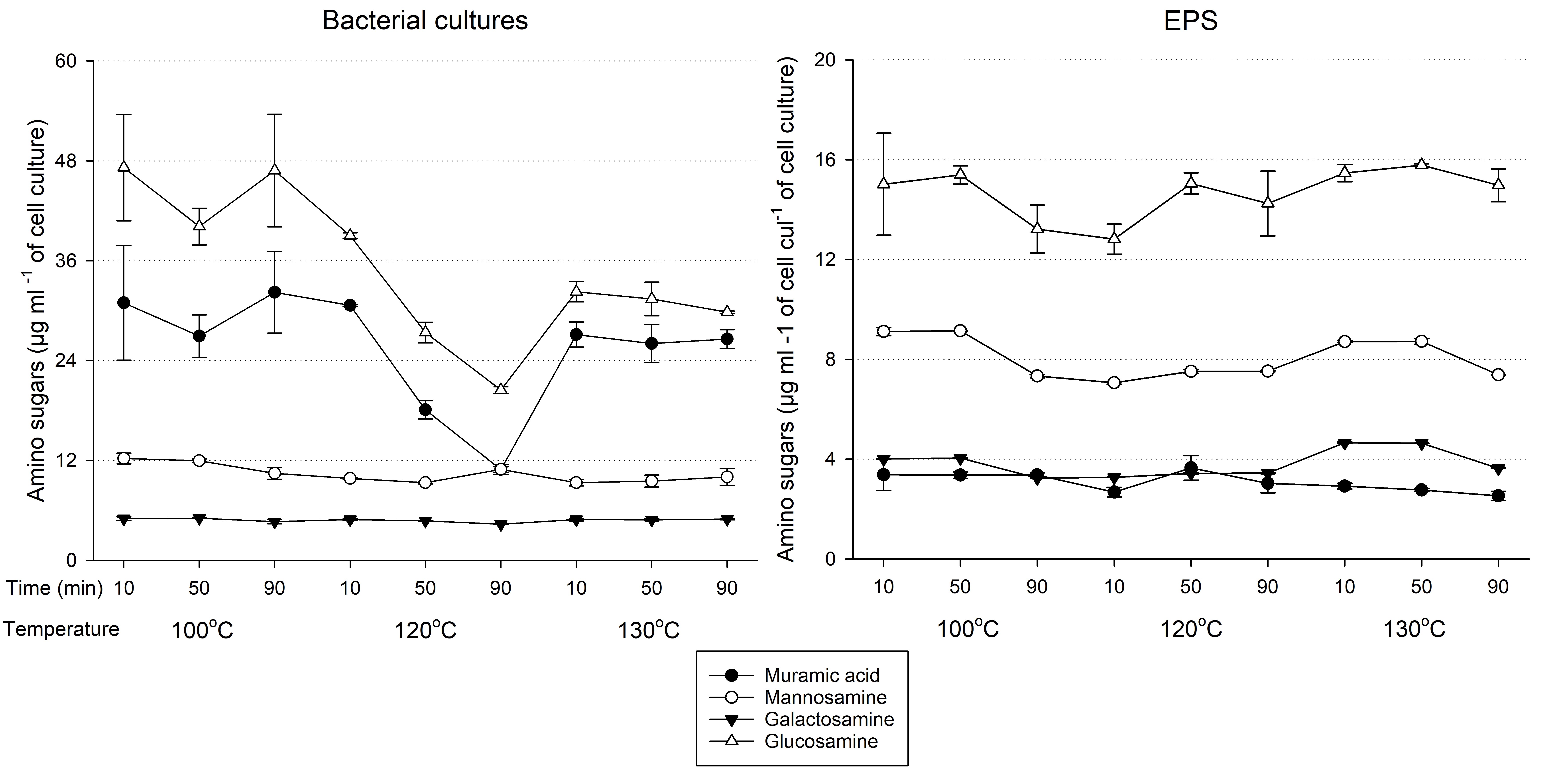

Supplement: Figure_S1_ycae038 [file figure_s1_ycae038.jpeg]

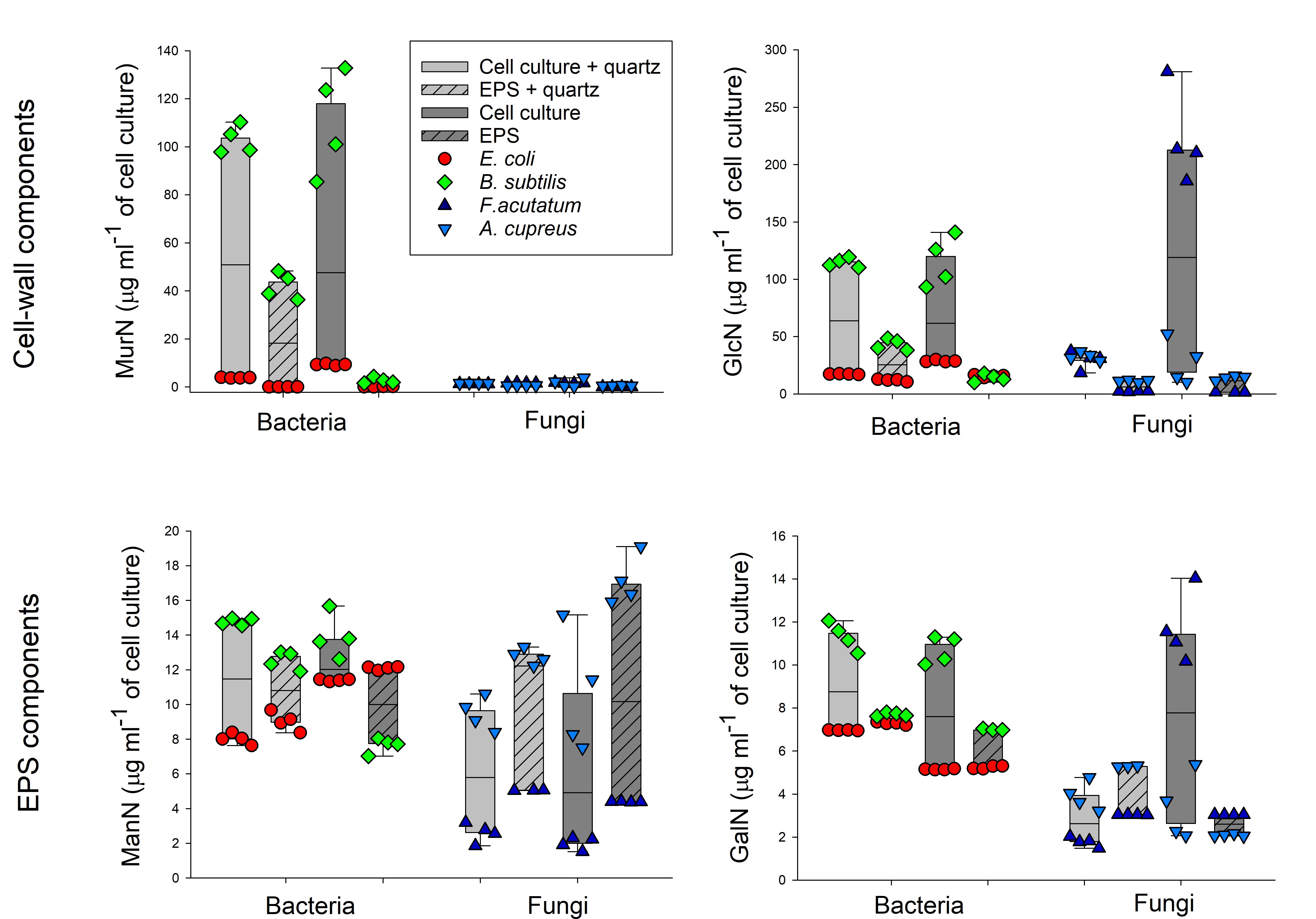

Supplement: Figure_S2_ycae038 [file figure_s2_ycae038.jpeg]
