# Supplementary material for: Galactosamine and mannosamine are integral parts of bacterial and fungal extracellular polymeric substances
Source: ISME Commun. 2024 Mar 22;4(1):ycae038. doi: 10.1093/ismeco/ycae038 (PMC11014887; doi:10.1093/ismeco/ycae038)
Supplement: Table_S1_ISME_16_2_24_ycae038 [file table_s1_isme_16_2_24_ycae038.docx]

Table S1. Quantified amounts of the four AS in bacterial cultures and EPS samples, following hydrolysis in the autoclave in different temperatures and times

| Temperature (°C) | Time (min) | Bacterial culture | | | | EPS | | | |
| --- | --- | --- | --- | --- | --- | --- | --- | --- | --- |
|  |  | MurN | ManN | GalN | GlcN | MurN | ManN | GalN | GlcN |
|  |  | (µg ml^-1^ of cell culture) | | | | | | | |
| 100 | 10 | 31.0 | 12.2 | 5.0 | 47.2 | 3.4 | 9.1 | 4.0 | 15.0 |
|  | 50 | 27.0 | 12.0 | 5.1 | 40.1 | 3.4 | 9.1 | 4.0 | 15.4 |
|  | 90 | 32.2 | 10.5 | 4.7 | 46.8 | 3.4 | 7.3 | 3.2 | 13.2 |
| 120 | 10 | 30.7 | 9.8 | 4.9 | 39.0 | 2.7 | 7.1 | 3.3 | 12.8 |
|  | 50 | 18.1 | 9.3 | 4.7 | 27.4 | 3.6 | 7.5 | 3.4 | 15.1 |
|  | 90 | 10.9 | 10.9 | 4.4 | 20.5 | 3.0 | 7.5 | 3.4 | 14.3 |
| 130 | 10 | 27.1 | 9.3 | 4.9 | 32.3 | 2.9 | 8.7 | 4.6 | 15.5 |
|  | 50 | 26.1 | 9.5 | 4.9 | 31.4 | 2.8 | 8.7 | 4.6 | 15.8 |
|  | 90 | 26.6 | 10.0 | 5.0 | 29.7 | 2.5 | 7.4 | 3.6 | 15.0 |
| Probability values | | |  |  |  |  |  |  |  |
| Temperature | | <.01 | <.01 | NS | <.01 | .02 | <.01 | <.01 | .05 |
| Time | | .01 | NS | .03 | <.01 | NS | <.01 | <.01 | NS |
| Temperature × Time | | <.01 | .05 | NS | <.01 | NS | <.01 | <.01 | NS |
| CV (± %) | | 8 | 5 | 2 | 6 | 8 | 1 | 0.3 | 5 |

CV = mean coefficient of variation between replicates in % (n = 4)
