# Supplementary material for: Galactosamine and mannosamine are integral parts of bacterial and fungal extracellular polymeric substances
Source: ISME Commun. 2024 Mar 22;4(1):ycae038. doi: 10.1093/ismeco/ycae038 (PMC11014887; doi:10.1093/ismeco/ycae038)
Supplement: Table_S2_ISME_16_2_24_ycae038 [file table_s2_isme_16_2_24_ycae038.docx]

Table S2. AS amounts quantified in the extracted EPS per treatment with two different hydrolysis methods (oven vs. autoclave). Results are expressed in µg ml^-1^ of cell culture after 4-day incubation and CV values represent the mean coefficient of variation between replicates (n=4).

| Treatment | Hydrolysis | MurN | ManN | GalN | GlcN |
| --- | --- | --- | --- | --- | --- |
|  |  | (µg ml^-1^ of cell culture) | | | |
| Quartz + Glycerol | Oven | 2.8 | 11.5 | 6.3 | 14.6 |
|  | Autoclave | 2.5 | 9.6 | 8.9 | 16.7 |
| CV (±%) |  | 13 | 12 | 11 | 19 |
| Glycerol | Oven | 2.8 | 16.5 | 9.3 | 27.3 |
|  | Autoclave | 2.6 | 11.7 | 9.4 | 28.8 |
| CV (±%) |  | 27 | 11 | 18 | 18 |
| Quartz + Starch | Oven | 0.9 | 10.8 | 5.4 | 9.9 |
|  | Autoclave | 0.9 | 11.3 | 5.6 | 12.6 |
| CV (±%) |  | 15 | 15 | 25 | 29 |
| Starch | Oven | 0.7 | 10.0 | 5.0 | 9.8 |
|  | Autoclave | 0.6 | 9.4 | 6.1 | 10.8 |
| CV (±%) |  | 21 | 12 | 20 | 18 |
| Probability values | |  |  |  |  |
| Species |  | NS | NS | NS | NS |
| Treatment |  | <.01 | <.01 | <.01 | <.01 |
| Hydrolysis |  | NS | NS | NS | .03 |
| Species x Treatment | | .03 | .02 | NS | NS |
| Species x Hydrolysis | | NS | NS | NS | NS |
| Treatment x Hydrolysis | | NS | .01 | NS | NS |
