# Supplementary material for: Galactosamine and mannosamine are integral parts of bacterial and fungal extracellular polymeric substances
Source: ISME Commun. 2024 Mar 22;4(1):ycae038. doi: 10.1093/ismeco/ycae038 (PMC11014887; doi:10.1093/ismeco/ycae038)
Supplement: Table_S3_ISME_16_2_24_ycae038 [file table_s3_isme_16_2_24_ycae038.docx]

| Treatment | Hydrolysis | MurN | | ManN | | GalN | | GlcN | |
| --- | --- | --- | --- | --- | --- | --- | --- | --- | --- |
|  |  | (µg ml^-1^ of cell culture) | | | | | | | |
|  |  | *M. luteus* | *P. fluorescens* | *M. luteus* | *P. fluorescens* | *M. luteus* | *P. fluorescens* | *M. luteus* | *P. fluorescens* |
| Quartz + Glycerol | Oven | 3.3 | 2.4 | 11.5 | 11.5 | 7.2 | 5.3 | 16.5 | 12.7 |
|  | Autoclave | 3.3 | 1.6 | 9.3 | 9.9 | 11.3 | 6.6 | 18.6 | 14.7 |
| Glycerol | Oven | 2.4 | 3.2 | 12.0 | 21.1 | 8.8 | 9.9 | 23.5 | 31.1 |
|  | Autoclave | 1.9 | 3.3 | 10.4 | 12.9 | 7.8 | 11.0 | 25.3 | 32.4 |
| Quartz + Starch | Oven | 1.1 | 0.7 | 11.8 | 9.9 | 5.7 | 5.0 | 9.1 | 10.7 |
|  | Autoclave | 0.9 | 0.9 | 11.8 | 10.8 | 5.2 | 6.0 | 14.0 | 11.2 |
| Starch | Oven | 0.7 | 0.7 | 10.1 | 9.8 | 5.1 | 5.0 | 10.0 | 9.6 |
|  | Autoclave | 0.6 | 0.7 | 9.3 | 9.6 | 6.0 | 6.2 | 11.0 | 10.7 |
| CV (±%) |  | 24 | 14 | 14 | 12 | 19 | 18 | 24 | 18 |

Table S3. AS amounts quantified in extracted EPS of two bacterial species grown under different substrate and matrix treatments. Results are expressed in µg ml^-1^ of cell culture after 4-day incubation and CV represents the coefficient of variation between replicates (n=4).
